# Stormwater Runoff Pollutant Loading Distributions and Their Correlation with Rainfall and Catchment Characteristics in a Rapidly Industrialized City

**DOI:** 10.1371/journal.pone.0118776

**Published:** 2015-03-16

**Authors:** Dongya Li, Jinquan Wan, Yongwen Ma, Yan Wang, Mingzhi Huang, Yangmei Chen

**Affiliations:** 1 College of Environment and Energy, South China University of Technology, Guangzhou, 510006, China; 2 The Key Lab of Pollution Control and Ecosystem Restoration in Industry Clusters, Ministry of Education, Guangzhou, 510006, China; 3 State Key Laboratory of Pulp and Paper Engineering, South China University of Technology, Guangzhou, 510640, China; Utah State University, UNITED STATES

## Abstract

Fast urbanization and industrialization in developing countries result in significant stormwater runoff pollution, due to drastic changes in land-use, from rural to urban. A three-year study on the stormwater runoff pollutant loading distributions of industrial, parking lot and mixed commercial and residential catchments was conducted in the Tongsha reservoir watershed of Dongguan city, a typical, rapidly industrialized urban area in China. This study presents the changes in concentration during rainfall events, event mean concentrations (*EMCs*) and event pollution loads per unit area (*EPLs*). The first flush criterion, namely the mass first flush ratio (*MFFn*), was used to identify the first flush effects. The impacts of rainfall and catchment characterization on *EMCs* and pollutant loads percentage transported by the first 40% of runoff volume (*FF_40_*) were evaluated. The results indicated that the pollutant wash-off process of runoff during the rainfall events has significant temporal and spatial variations. The mean rainfall intensity (*I*), the impervious rate (*IMR*) and max 5-min intensity (*I_max5_*) are the critical parameters of *EMCs*, while *I_max5_,* antecedent dry days (*ADD*) and rainfall depth (*R_D_*) are the critical parameters of *FF_40_*. Intercepting the first 40% of runoff volume can remove 55% of *TSS* load, 53% of *COD* load, 58% of *TN* load, and 61% of *TP* load, respectively, according to all the storm events. These results may be helpful in mitigating stormwater runoff pollution for many other urban areas in developing countries.

## Introduction

Many catchment areas are undergoing fast urbanization and industrialization in developing countries due to rises in population and economic growth, and these processes have significant influences on the quality of urban stormwater runoff [1, 2].

Urban stormwater runoff, which degrades streams by changing the volume, pattern and quality of flow, presents a problem that challenges dominant approaches to storm and water resource management, as well as to environmental flow assessment [3, 4]. The characteristics of stormwater runoff quality, hydrology, retention and other issues have all been examined in the literature, and it has been found that significant quantities of organics, nutrients, and heavy metals are present in stormwater runoff [5–8]. In addition, nonpoint source (NPS) pollution due to urban stormwater runoff is considered as one of the major causes of water-related adverse health impacts among urban residents [9].

It has long been recognized that the pollutant build-up and wash-off processes are influenced by rainfall and catchment characteristics [10, 11]. Stormwater runoff pollution is a very serious problem, and the temporal and spatial variations in this pollution process can be quite significant in rapidly industrialized cities, because fast urbanization and industrialization are usually characterized by an increase in the number of factories and population density, as well as the drastic changes in land-use, moving from farmland and green land to impervious surfaces [12]. Numerous efforts have been made to investigate the relationships between stormwater runoff pollution and rainfall characteristics for various catchment areas, such as residential, commercial, and industrial areas, as well as highways, parking lots, bridges and roofs [4, 13, 14]. However, it is difficult to identify the characteristics of stormwater runoff from such catchments because of the mixed land-use types, slow development of sewage treatment infrastructure, and poor waste management in rapidly industrialized cities [2]. There are thus very few studies which report the stormwater runoff characteristics of such catchments in rapidly industrialized urban areas in developing countries with a high population density (e.g., China). This lack of research means that little is known about the mechanisms underlying urban stormwater runoff pollutant transport and the influence of rainfall, as well as various catchment characteristics, on the pollutant loading of rapidly industrialized cities. Understanding these interactions would be useful for improving design criteria and strategies for controlling urban stormwater runoff pollution. There is thus a need to characterize and examine stormwater runoff quality and pollutant loading, as well as their correlations with rainfall and catchment characteristics, in a rapidly industrialized city, in order to improve management in this area.

A total of 10 rain events were surveyed at industrial, parking lot and mixed commercial and residential catchments in the rapidly industrialized Tongsha reservoir catchment in China, during the period from April 2009 to September 2011. The stormwater runoff and quality parameters were analyzed to assess the temporal characteristics of stormwater runoff with different kinds of land-use. The objectives of this study were as follows: (1) to characterize the temporal variations in pollutant wash-off during rain events and the spatial variations with regard to different land-use catchments; (2) to identify the first flush phenomena using *MFFn*; and (3) to use the results of this work with regard to the runoff pollution load distributions and the main factors to improve runoff management schemes in rapidly industrialized urban areas.

## Materials and Methods

### Study Area

**Fig 1:**
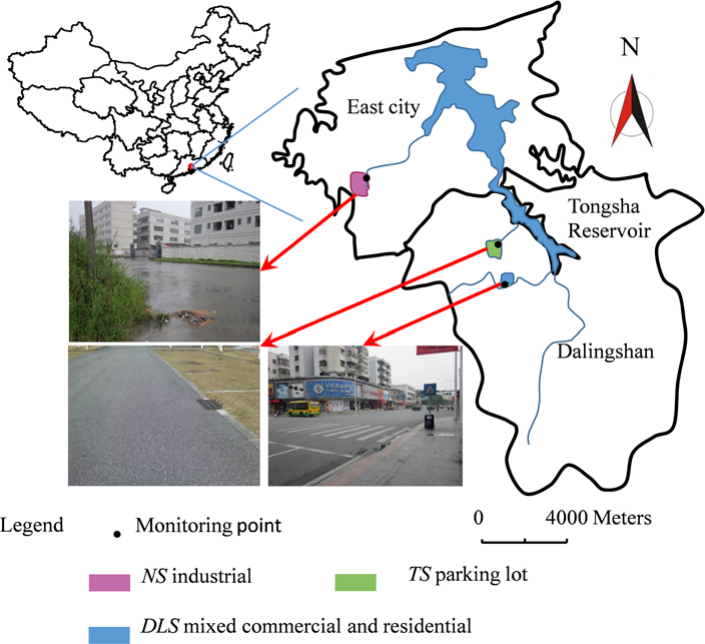
Study sites.

This study was conducted in the Tongsha catchment in Dongguan City, Southeast China (Fig. 1), and was approved by the Dongguan Government of Guangdong Province. No specific permissions were required to study these locations, as they do not involve endangered or protected species. The GPS coordinates of the study areas are as follows: Niushan (*NS*) industrial zone, 22°57′23.63″N, 113°46′17.63″E. Dalingshan (*DLS*) mixed commercial and residential area, 22°54′09.32″N, 113°50′24.10″E. Tongsha (*TS*) parking lot, 22°55′55.79″N, 113°48′00.37″E."

The total drainage area is 100 km^2^, including two regions of Dalingshan and East City. The climate is a typical subtropical monsoon climate, with a mean annual temperature of 22.5°C and mean annual precipitation of 1790 mm. The rain mostly occurs during the period from April to September, due to the impact of monsoons and typhoons.

As one of the largest global manufacturing bases, Dongguan City has factories that operate in many important industries. While this has helped in the development of the economy, it was led to serious water pollution, especially with regard to stormwater runoff pollutants. Tongsha catchment is located on the west bank of the Tongsha reservoir, which is currently suffering from severe eutrophication caused by the urban stormwater runoff pollution.

Tongsha reservoir catchment was selected as the study area in this work, and divided into three land-use categories (industrial, parking lot, and mixed commercial and residential catchments). Based on the land-use characteristics of the runoff watershed and the related physiographic factors, the Tongsha reservoir catchment was divided into three watersheds, namely the Niushan (*NS*) industrial area, the Dalingshan (*DLS*) mixed commercial and residential area, and the Tongsha (*TS*) parking lot catchment. A brief summary of the physical characteristics of these three watersheds is given in Table 1. There are seven sewer sub-catchments in Tongsha reservoir catchment, three in *NS* industrial catchment, two in *DLS* mixed commercial and residential catchment, two in *TS* parking lot catchment. The sampling sites were on the total sewer drains of *NS*, *DLS* and *TS* as shown in Fig. 1.

**Table 1:** Basic characteristics of the monitoring sites.

watershed	NS	DLS	TS
Monitor location	Niushan (*NS*) industrial zone	Dalingshan (*DLS*) mix of commercial and residential	Tongsha (*TS*) parking lot
Drainage area	6.89 ha	3.64 ha	5.12 ha
Percentage of impervious area	74%	87%	37%
Sewer type	Separated	Separated	Separated
Primary land use	Industrial	Commercial	Greening
Land slope	2.80%	1.10%	1.50%

### Runoff Sampling and Analysis

Field experiments for 16 non-rainy days and 10 storm events between April 2009 and September 2011 were conducted synchronously at the three experimental watersheds. Sampling was started at the initiation of the rain event and ended when the flow receded down to the dry weather water level. The sampling was generally done at 5 to 15 min intervals when the flow was rising, and then at 20 to 60 min intervals for the receding flow. 9 to 14 samples were collected to assess the water quality for each storm event. All the samples were collected manually by sampling at a depth of 10 cm from the water surface, and stored in 1.0 L glass sample bottles with Teflon lined screw caps. Three identical water samples were taken at each sampling to ensure precision and accuracy. The samples were refrigerated and analyzed within 8 h after collection. The typical water quality pollutant parameters were measured, including total suspended solids (*TSS*), chemical oxygen demand (*COD*), total nitrogen (*TN*), ammonia nitrogen (*NH*_*4*_^*+*^ *-N*), total phosphorus (*TP*), and heavy metals (*Fe*, *Zn*, *Cu*). The samples were treated according to the standard procedures [15].

Rainwater was also collected for comparative analysis, because rainfall has the potential to entrain atmospheric pollutants. In addition, the flow rates and flow volumes corresponding to each sample were monitored and computed. In *NS*, *DLS* and *TS*, the flow rates were measured by propeller flow meters. The water stage was recorded by a water level gauge at 5 min intervals at all the monitoring sites. Recording rain gauges (*MDZH4T1601*) near the monitoring sites simultaneously recorded the characteristics of 10 rain events from April 2009 to September 2011. The characteristics of the monitored rainfall events are shown in Table 2. Three representative rainfall events (15/4/2009, 58.80 mm, 20/10/2009, 9.36 mm, 20/10/2010, 22.50 mm) were selected for further analysis.

**Table 2:** Characteristics of the rainfall events (*n* = 10).

*Date*	*R*_*D*_ *(mm)*	*R*_*Dur*_ *(min)*	*I (mm/h)*	*I*_*max5*_ *(mm/min)*	*ADD (d)*	*Sample number*
15/4/2009	58.80	210	16.8	1.32	64	14
15/9/2009	9.75	65	9	0.66	4	9
20/10/2009	9.36	104	5.4	0.56	15	10
2/6/2010	33.60	210	9.6	0.58	3	14
28/6/2010	41.60	130	19.2	0.8	16	14
20/10/2010	22.50	150	9.1	0.34	21	14
16/4/2011	88.40	260	20.4	1.56	46	14
3/5/2011	8.40	127	4.2	0.54	15	10
9/8/2011	17.69	131	8.1	1.61	5	11
21/9/2011	15.60	52	18	0.71	8	10

*I*_*max5*_: Max 5-min intensity,

*I*: Mean rainfall intensity,

*R*_*Dur*_: Rainfall duration,

*R*_*D*_: Rainfall depth,

*ADD*: Antecedent dry days.

### Data Processing and Statistical Analysis

Event mean concentration (*EMC*) is a key analytical parameter, which refers to a flow-weighted average concentration in the whole process of a rainfall-runoff event, defined as the total pollution load mass divided by the total runoff volume [16], and this can be used to evaluate the effects of rainfall runoff on the water quality of the receiving waters. The value of *EMC* is expressed as:

$$EMC=\frac{M}{V}=\frac{\int_{0}^{t}C_{t}Q_{t}dt}{\int_{0}^{t}Q_{t}dt}≅\frac{\sum C_{t}Q_{t}\Delta t}{\sum Q_{t}\Delta t}$$ (1)

Event pollution load per unit area (*EPL*), refers to the amount of pollutants emitted per unit area in the whole rainfall event. It can be expressed as:

$$EPL=\frac{M}{A}=\frac{\int_{0}^{t}C_{t}Q_{t}dt}{A}≅\frac{\sum C_{t}Q_{t}\Delta t}{A}$$ (2)

For each storm and water quality parameter, the magnitude of the first flush can be quantified by using a mass first flush ratio (*MFFn*) [14]. The *MFFn* is a useful index, because it means that various storm and water quality data can be optimized and used as indicators in statistical analysis. *MFFn* can be calculated for any point in a storm, and is defined as follows:

$$MFFn=\frac{\int_{0}^{t_{i}}C_{t}Q_{t}dt/M}{\int_{0}^{t_{i}}Q_{t}/V}≅\frac{\sum_{t=0}^{t=t_{i}}C_{t}Q_{t}\Delta t/M}{\sum_{t=0}^{t=t_{i}}Q_{t}\Delta t/V}$$ (3)

Where *n* is the index or point in the storm, which corresponds to the percentage of the runoff, ranging from 0% to 100%; *M* (g) is the pollutant mass during the rainfall event; *V* (m^3^) is the runoff volume during the rainfall event; *C*_*t*_ (mg/L) is the pollutant concentration at time *t*; *Q*_*t*_ (m^3^/s) is the discharge runoff flow rate at time *t*; *t* refers to the time of total runoff; *t*_*i*_ is the time up to point *n* in the event, and *△t* is the interval time of sampling. Finally, *A* (km^2^) is the catchment area.

The *EMCs*, *EPLs* and *MFFn* of the runoff pollutants were calculated to describe the characteristics of the pollutant output process regulations. The Kolmogorov-Smirnov test was carried out on a single sample to check out the normal distributions of key parameters such as the *EMCs* and *EPLs* of the runoff pollutants and rainfall variables, and to ensure that the basic assumptions of the Pearson correlation analysis were met. The statistical analysis software packages *SPSS 17*.*0* and *Origin 8*.*0* were used to compute the Pearson correlation coefficients and principal component analysis to determine the correlations among the *EMCs* and *FF*_*40*_ of the five pollutants and the explanatory variables of the rainfall events and catchment areas. Multiple linear regression analysis was employed to determine the relationship between the storm pollution loads of *FF*_*40*_ (*|FF*_*40*_ *| = EPLs*×*FF*_*40*_) and storm characteristics as in Eq. (4)

$$\left|FF_{40}\right|=a\pm b\left(R_{D}\right)\pm c\left(R_{Dur}\right)\pm d\left(I\right)\pm e\left(I_{\max5}\right)\pm f\left(ADD\right)$$ (4)

Where *a* is arbitrary constant and *b*, *c*, *d*, *e* and *f* are coefficients for each rainfall variable.

## Results and Discussion

### Characteristics of Pollutant Wash-Off

**Fig 2:**
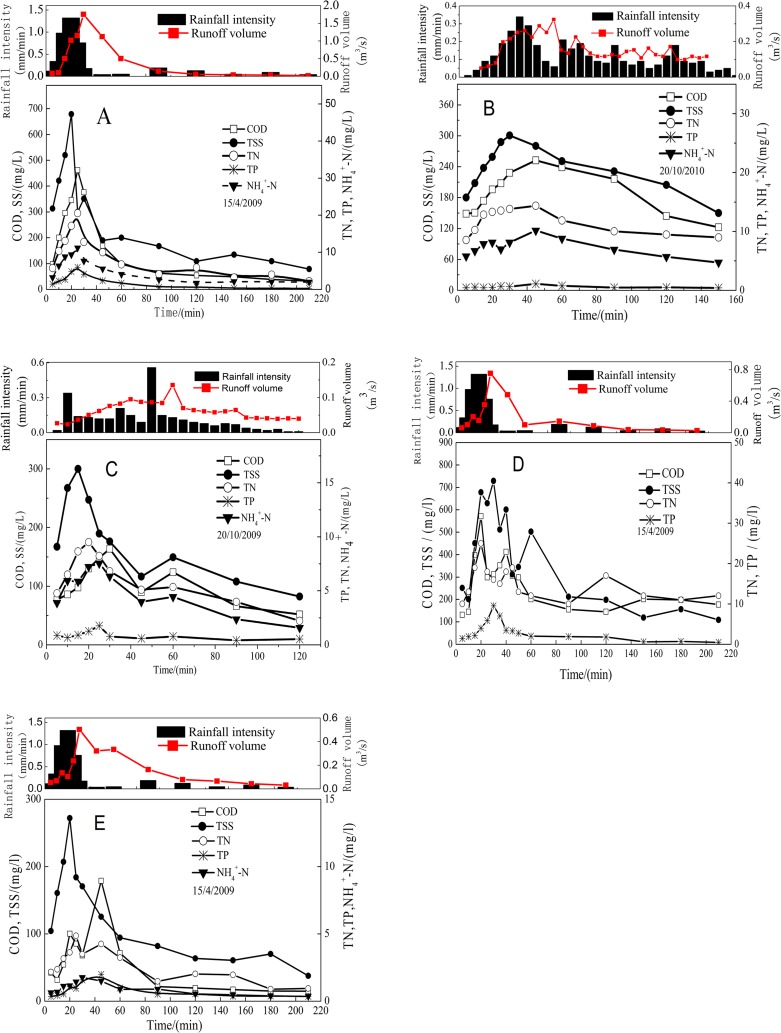
Pollutant wash-off process curves in *NS*, *DLS* and *TS*.

Ten rain events were monitored at the *NS* industrial, *TS* parking lot, and *DLS* mixed commercial and residential catchments during the period from 15/4/2009 to 21/9/2011. Table 2 summarizes the characteristics of the rain events and sites, such as event date, Max 5-min intensity (*I*_*max5*_), mean rainfall intensity *(I)*, rainfall duration *(R*_*Dur*_ *)*, rainfall depth *(R*_*D*_ *)*, Antecedent dry days (*ADD*), and sample number. The *R*_*D*_ varied from 8.40 to 88.40 mm and *ADD* from three to 64 days. *R*_*Dur*_ were measured from 52 to 260 min, and *I* were determined from 4.2 to 20.4 mm/h. Fig. 2A, B and C illustrates the data collected at the industrial watershed sampling sites during the three storm events of 15/4/2009 (58.80 mm), 20/10/2010 (22.50 mm) and 20/10/2009 (9.36 mm). These rainfall events differed, in that the event of 15/4/2009 had a single peak, and the event of 20/10/2009 had a bimodal peak, while the event of 20/10/2010 did not have any significant peak. A secondary flush effect phenomenon common to bimodal rainfall events occurred during the event of 20/10/2009. The pollutant concentrations peaked slightly after the rainfall intensity peak. During the event of 15/4/2009, the concentrations of *COD*, *TSS*, *TN*, *TP*, and *NH*_*4*_^*+*^ *-N* peaked shortly after the runoff was generated, which could be due to the fact that the relatively higher kinetic energy of high-intensity rainfall events results in more pollutants being transported [17]. Because of adequate flushing, the concentrations sharply declined to 1/3 ~ 1/5 of the peak values approximately 40 min after the runoff was generated, which is consistent with the findings of Kim (2007) [10]. However, the pollutant concentrations of the event of 20/10/2010 fell to less than 1/2 of the peak values at the end of the rainfall, and no significant first flush phenomenon was found. As shown in Fig. 2, the fall in concentrations of *COD*, *TSS*, *TN*, *TP*, and *NH*_*4*_^*+*^ *-N* were directly associated with the rainfall intensity during the three events. The results suggest that a higher rainfall intensity is likely to be associated with a higher first flush in the same watershed.

Fig. 2*A*, *D* and *E* present the variations in the *COD*, *TSS*, *TN*, *TP*, and *NH*_*4*_^*+*^ *-N* concentrations for the industrial, commercial and residential and parking lot areas during the same rainfall event of 15/4/2009. Most of the pollutants on the paved surfaces were washed off within 30 to 60 min of the storm beginning, although there were some variations among the five pollutant species and land-use patterns. Comparing the three watersheds, the highest pollutant concentration peaks were found in the mixed commercial and residential watershed. The pollutant concentrations decreased sharply after the initial stage of the peaks in the industrial watershed and parking lot, at about 40 and 60 min, respectively. However, the pollutant concentrations in the mixed commercial and residential watershed rose to their submaximal peak levels at 60 min, and then decreased slowly. Due to the differences in the catchment characteristics, the time intervals between the rainfall intensity peaks and pollutants concentration peaks during the three rainfall events were 10 min, 25 min, and 40 min, respectively. Furthermore, the characteristics of the wash-off process with regard to five pollutants showed some significant differences, even for the same storm events and watersheds. For example, *TSS* had the highest variations and peak concentrations during the storm events in the industrial watershed, whilst *TP* had the lowest. This means that the variability of the pollutants’ wash-off characteristics within the same storm event was significantly influenced by both pollutant species and watershed land-use.

### Spatial Variation of EPLs and EMCs

*EMCs* and *EPLs* in Tables A, B, C, D, E and F in S1 File were determined using *Eq*. (1) and (2), with the results summarized in Table 3. Most of the constituent concentrations in the rainwater were below the detection limits, and thus the rainwater quality was not influenced by the pollutant contents in the storm, except for *TN*. This implies that rainfall is a significant source of nitrogen in the urban catchment area. It can be seen that the *DLS* mixed residential and commercial catchment has the highest median *EMCs* and *EPLs* for *BOD*, *COD*, *TSS*, *NH*_*4*_^*+*^ *-N*, and *TP*, followed by the *NS* industrial and *TS* parking lot catchments. The main sources of organic matter during storm events are restaurants and food stalls, especially in the commercial catchment area. The *EMCs* values for *COD* and *TSS* of *DLS* mixed commercial and residential area were much higher than those in other areas with the same watershed features [4]. Comparing the runoff water quality with regard to *TN* and *TP*, the runoff concentration values in the *DLS* were more than 10 times higher than those found in the earlier studies [18]. The reason for this may be that this area is in the busiest part of the city, and thus sewage and food scraps, along with other trash, are dumped by shops and restaurants on both sides of the path. In addition, this area only tends to be swept once a day, due to poor environmental management.

**Table 3:** Basic statistics of runoff pollution *EMCs* and *EPLs* in all storm events.

*Type of land use*	*Statistics*	*COD*	*TSS*	*TN*	*TP*	*NH*_*4*_^*+*^ *-N*	*Fe*	*Zn*	*Cu*
*DLS* mix of commercial and residential *EMCs*	Mean	302.81	367.19	16.69	3.17	5.52	1.56	0.33	-------
	Standard deviation	151.35	173.04	12.88	2.22	2.66	0.95	0.15	-------
	Maximum	567.11	708.40	39.05	7.09	9.13	2.98	0.57	-------
	Minimum	103.25	141.03	3.49	0.42	1.35	0.12	0.11	-------
*NS* industrial zone *EMCs*	Mean	221.45	298.11	8.98	2.12	4.27	4.27	3.50	0.31
	Standard deviation	126.38	168.07	4.69	1.18	2.10	1.88	1.40	0.12
	Maximum	486.22	651.30	16.65	4.06	7.49	6.78	5.12	0.47
	Minimum	81.93	96.93	2.46	0.67	0.99	1.23	1.57	0.11
*TS* parking lot *EMCs*	Mean	67.94	86.72	2.33	1.02	0.85	0.37	-------	-------
	Standard deviation	32.04	30.71	1.11	1.21	0.40	0.24	-------	-------
	Maximum	122.21	133.09	4.34	4.05	1.36	0.85	-------	-------
	Minimum	17.18	25.80	0.91	0.09	0.12	0.11	-------	-------
*DLS* mix of commercial and residential *EPLs*	Mean	57.13	67.33	2.95	0.63	1.14	0.37	0.07	-------
	Standard deviation	52.20	61.53	2.53	0.63	1.22	0.46	0.08	-------
	Maximum	132.15	196.30	7.19	1.83	3.66	1.41	0.27	-------
	Minimum	8.08	10.59	0.26	0.03	0.10	0.01	0.01	-------
*NS* industrial zone *EPLs*	Mean	39.04	57.47	1.82	0.44	0.89	0.89	0.63	0.06
	Standard deviation	42.02	59.25	1.97	0.55	1.07	0.97	0.66	0.07
	Maximum	138.30	160.40	6.26	1.73	3.55	3.22	2.27	0.22
	Minimum	7.24	10.55	0.24	0.05	0.07	0.09	0.14	0.01
*TS* parking lot *EPLs*	Mean	8.18	10.97	0.26	0.10	0.11	0.05	-------	-------
	Standard deviation	7.98	10.14	0.24	0.11	0.10	0.07	-------	-------
	Maximum	24.35	30.33	0.78	0.29	0.28	0.24	-------	-------
	Minimum	1.09	1.16	0.07	0.01	0.01	0.01	-------	-------
Natural rainfall	*EMCs(mg/L)*	14.62	——-	0.98	0.43	0.46	-------	——-	-------

In contrast, the *NS* industrial catchment had the highest medians *EMCs* and *EPLs* for *Fe*, *Zn* and *Cu*. The *EMCs* and *EPLs* for metals at the *NS* industrial catchment were far greater than those for the mixed residential and commercial or parking catchments. The *Zn* could be from roofs, factories, and vehicle wear and tear in the industrial catchment. The large storm to storm variations of *EMCs* and *EPLs*, as well as the diverse kinds of land-use in urban catchments, mean that a long-term monitoring program is needed in order to better estimate the *EMCs* and *EPLs* values. The *EMCs* values of *COD*, *TN*, *TP* and *NH*_*4*_^*+*^ *-N* were close to those found in Lee [4]. The pollution loading levels found in the current study are also quite similar to those found for the Shiyan reservoir catchment in Shenzhen, China [1]. The average *EMCs* of *COD*, *TSS*, *TN* and *TP* in *TS* parking lot were 67.94 mg/l, 86.72 mg/l, 2.33 mg/l and 1.02 mg/l, respectively, more than two times of the values found in Japan except for *TSS* and *TP*. The values of *TSS* and *TP* are similar to the values of the study [19]. The *EMCs* and *EPLs* values of *TS* parking lot were much lower than that in *NS* and *DLS*, which could be the reason that grassland interception could reduce the contamination due to rainfall runoff, and the stormwater runoff pollution from grassland cannot be ignored.

### First Flush Effect Analysis Using MFFn

**Fig 3:**
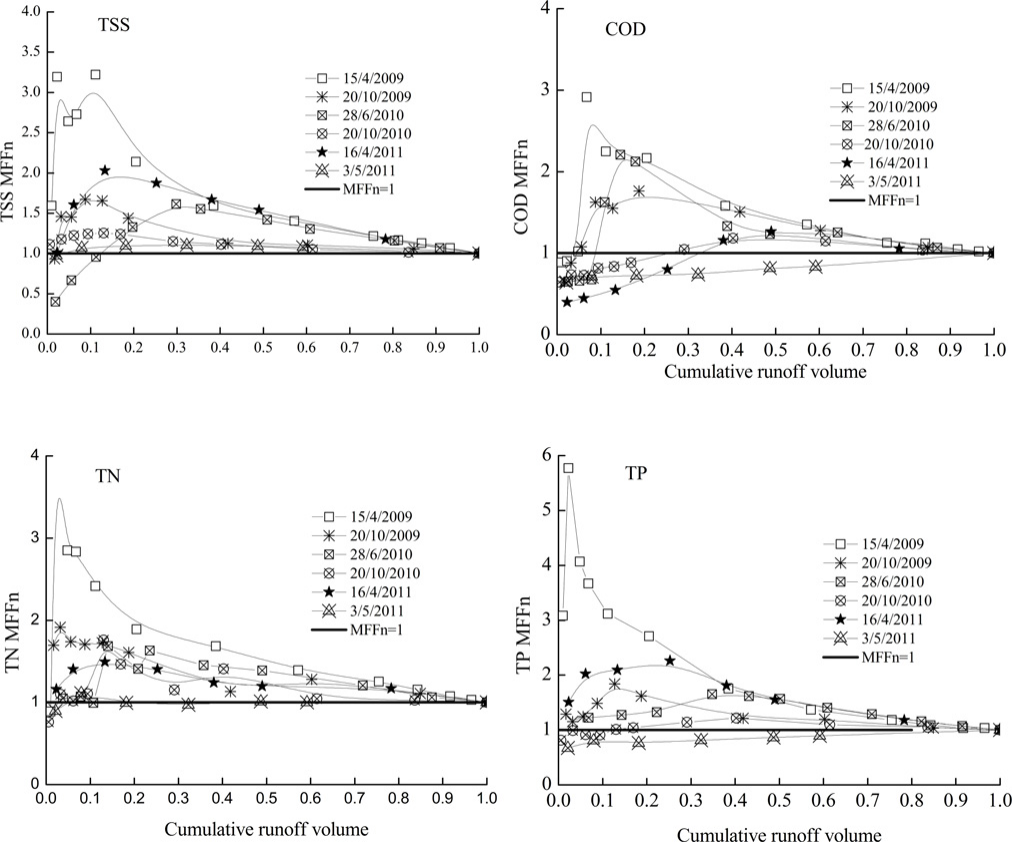
The indicator *MFFn* used to identify the first flush effect in *NS*.

**Fig 4:**
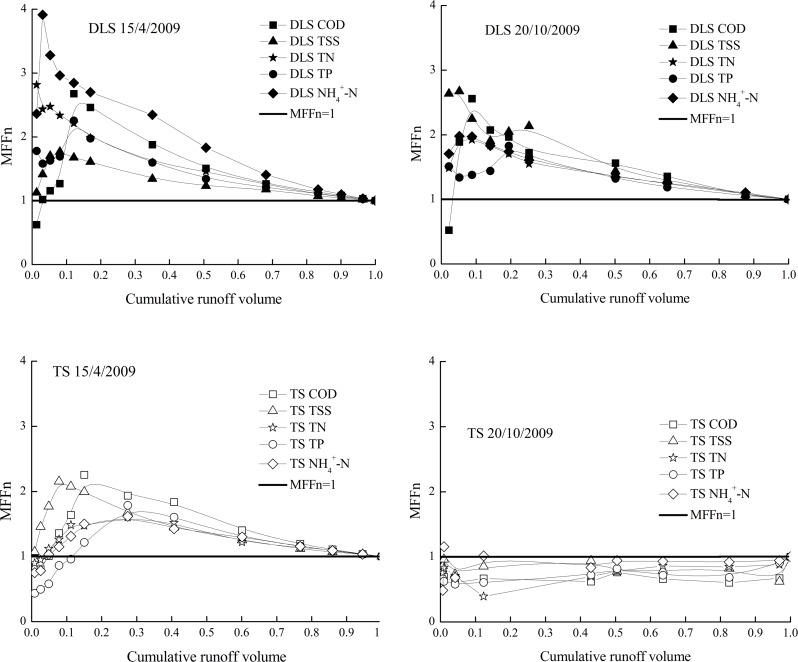
The indicator *MFFn* used to identify the first flush effect in *DLS* and *TS*.

The first flush effects of all the quality parameters were studied by plotting the *MFFn* against the cumulative runoff volume, as shown in Figs. 3 and 4. The first flush was observed when the data ascended above the balanced line (*MFFn* = 1). The balanced line (*MFFn* = 1) represents when the concentration of pollutants remained constant throughout the stormwater runoff. Conversely, dilution was assumed to have occurred when the data fell below the balanced line (*MFFn* = 1). The deviation of the cumulative pollutant mass curve from the balanced line (*MFFn* = 1) was used as a measure of the strength of the first flush. The deviation is positively related to the first flush coefficient *MFFn*.

In the current study, most of the *TSS*, *COD*, *TN*, and *TP MFFn* curves exceeded the balanced line (*MFFn* = 1) in the *NS* industrial catchment, except for the event of 3/5/2011, meeting Geiner’s definition of a first flush [20]. The first flush is greatest for the event of 15/4/2009, although it falls short of fitting Bertrand-Krajewski et al.’s (1998) definition of a first flush (only 67% of the mass, as opposed to 80%, was discharged in the first 30% of the runoff volume) [16].

When comparing the *MFFn* values for the six events at the *NS* industrial watershed, the event of 15/4/2009 showed strongest first flush for all constituents, and the first flush relative strength of most of the constituents is in accordance with the max 5-min intensity (*I*_*max5*_). This indicates that the strength of the first flush is in proportion to *I*_*max5*_, *I*, *ADD* and *IMR*. Additionally, it is notable that the *MFFn* values of the pollutant species also vary based on the rainfall characteristics along the cumulative runoff volume axis, which confirms that different rainfall characteristics could lead to the different stormwater first flush characteristics of various pollutant species.

The event of 3/5/2011 had the lowest values of *MFFn* for all the pollutants in *NS* industrial zone. This is probably due to the small *I*_*max5*_ (0.54 mm/min) and *I* (4.2 mm/h), and the limited *R*_*D*_ (8.40 mm) of the event. Low max 5-min and mean intensity rainfall, as within this event, typically leads to a weak first flush, because the runoff flow does not have sufficient energy to scour and mobilize the pollutants, and the limited rainfall depth may not have sufficient runoff volume to wash out the pollutants, meaning that a decline in the pollutant concentrations can be observed near the end of the storm, as explained by Kang et al. (2006) [21]. The entire runoff volume of a small storm, such as the event of 3/5/2011, can be less than the first flush volume seen with most large storms, and thus the lack of a first flush for a small storm may not be a disadvantage when using *BMPs* optimized for treating the first flush.

As shown in Fig. 3, the relative strength of the first flush with regard to the various pollutants is *TP>TSS>TN>COD* for the *NS* industrial watershed, while it is *NH*_*4*_^*+*^ *-N>TN>COD>TSS>TP* for the *DLS* mixed commercial and residential watershed, as shown in Fig. 4. Finally, the relative strength of the first flush with regard to the various pollutants is *TSS > COD >TN> NH*_*4*_^*+*^ *-N>TP* for the *TS* parking lot. *TN* and *COD* had larger first flushes in the *DLS* mixed commercial and residential watershed than in the *NS* industrial watershed, and this was also observed by Lee et al. (2002) [22].

### Runoff Pollution Load Distributions

Based on the results of dimensionless accumulative analysis, the *FF*_*30*_, *FF*_*40*_, *FF*_*50*_ and *FF*_*60*_ of the main pollutants were calculated during the 10 events, and the results are given in Table 4. The mean values of the *FF*_*40*_ for *TSS*, *COD*, *TN*, *TP* were 55%, 53%, 58%, 61%, respectively. More than 83% of the pollution load mass was delivered in the initial 60% of the runoff volume, which exhibited a greater first flush effect than that reported by Bertrand-Krajewski et al. (1998). This earlier study found that 80% of the total pollutant mass was transported in the first 74% of the total volume in separate sewer systems, and 80% of the total pollutant mass was transported in the first 79% of the total volume in the combined sewer system [16].

**Table 4:** Statistical summary of *FF*_*30*_, *FF*_*40*_, *FF*_*50*_ and *FF*_*60*_ for *TN*, *TP*, *COD* and *TSS*.

	*TSS*				*COD*				*TN*				*TP*			
	*FF*_*30*_	*FF*_*40*_	*FF*_*50*_	*FF*_*60*_	*FF*_*30*_	*FF*_*40*_	*FF*_*50*_	*FF*_*60*_	*FF*_*30*_	*FF*_*40*_	*FF*_*50*_	*FF*_*60*_	*FF*_*30*_	*FF*_*40*_	*FF*_*50*_	*FF*_*60*_
Mean	45%	55%	75%	83%	38%	53%	69%	87%	34%	58%	79%	89%	41%	61%	64%	84%
Maximum value	57%	66%	80%	89%	56%	69%	84%	91%	52%	80%	83%	94%	68%	79%	84%	92%
Minimum value	31%	32%	62%	73%	24%	37%	54%	81%	21%	32%	58%	76%	25%	31%	53%	69%
Standard deviation	15%	9%	3%	2%	12%	8%	8%	7%	17%	14%	8%	7%	22%	13%	15%	12%

In addition, comparing the results from *FF*_*60*_, *FF*_*50*_, *FF*_*40*_ and *FF*_*30*_, the *FF*_*40*_ interception of the first 40% of the total runoff volume could remove an average of 10%, 15%, 24% and 20% of the pollution loads for *TSS*, *COD*, *TN* and *TP*, respectively, more than that intercepted by 30% of the total runoff volume. An average of 58% *TN* and 61% *TP* of the *EPLs* could be removed by intercepting the initial 40% of the runoff volume, which is a critical measure with regard to controlling the eutrophication of urban water bodies. It is thus suggested that *FF*_*40*_ should be adopted as the interception ratio of the initial stormwater runoff volume in the Tongsha reservoir watershed.

### Correlation between Rainfall and Catchment Parameters with EMCs and FF_40_

The relationships between the *EMCs* and *FF*_*40*_ of various constituents with the various storm and watershed characteristics were analyzed by Pearson correlation analysis (Table 5) (See Table G, H and I in S1 File). The storm characteristics included *I*_*max5*_, *I*, *R*_*Dur*_, *R*_*D*_ and *ADD* [23, 24]. The watershed characteristics included the Catchment Area *(CA)* and *IMR*.

**Table 5:** Correlations between *EMCs*, *FF*_*40*_, rainfall and catchment characteristics.

	*The characteristic parameters of rainfall*						*Catchment characteristics*	
*Parameter*	*Type of pollutants*	*I*_*max5*_	*I*	*ADD*	*R*_*Dur*_	*R*_*D*_	*CA*	*IMR*
*EMC*	*COD*	0.015	0.417*	0.058	-0.115	0.115	-0.19	0.66**
	*TSS*	0.041	0.395*	0.088	-0.169	0.076	-0.12	0.67**
	*TN*	0.414*	0.402*	0.167	0.124	0.297	-0.30	0.58**
	*TP*	0.271	0.466**	0.101	-0.067	0.157	-0.22	0.48**
*FF*_*40*_	*COD*	0.462*	0.34	0.462*	0.34	0.454*	-0.10	0.02
	*TSS*	0.486*	0.341	0.286	0.12	0.332	0.05	0.25
	*TN*	0.416*	0.319	0.377*	0.273	0.415*	-0.35	-0.10
	*TP*	0.394*	0.237	0.24	0.223	0.351	-0.16	0.20

*I*_*max5*_: Max 5-min intensity,

*I*: Mean rainfall intensity,

*R*_*Dur*_: Rainfall duration,

*R*_*D*_: Rainfall depth,

*ADD*: Antecedent dry days,

*CA*: Catchment area,

*IMR*: Impervious rate.

**: *P* values<0.01,

*: *P* values <0.05.

Based on an analysis of the correlation matrix, the most important parameters with regard to the *EMCs* of various pollutants are *I*_*max5*_, *I* and *IMR*. The strongest correlations were found for *IMR* and the *EMCs* of *COD* (*r = 0*.*66*), *TSS* (*r = 0*.*67*), *TN* (*r = 0*.*58*) and *TP* (*r = 0*.*48*) at *P* <0.01 significance levels, with results that were very similar to the influence of the impervious area fraction on pollutant wash-off [11]. *I* was positively correlated with the *EMCs* of *COD* (*r = 0*.*417*, *P<0*.*05*), *TSS* (*r = 0*.*395*, *P<0*.*05*), *TN* (*r = 0*.*402*, *P<0*.*05*) and *TP* (*r = 0*.*466*, *P<0*.*05*). A larger mean rainfall intensity led to an increase in flush intensity of contaminants on the surface. Elsewhere, *I* was also found to be positively correlated with *EMCs* of stormwater runoff pollutant [1, 9, 25]. The positive correlation was also found between *I*_*max5*_ and the *EMCs* of *TN* at a *P<0*.*05* significance level, because of a complex *TN* source in the rapidly industrialized city due to *poor litter management* and bare land. It was thought that *TN* source depletion and pollutant dilution would not happen in the catchment. Other variables, including the rainfall characteristic parameters (*ADD*, *R*_*Dur*_, and *R*_*D*_) and *CA* did not display obvious correlations with the *EMCs* of the pollutants (*P>0*.*05*), indicating that these parameters had no apparent influence on the *EMCs* in all three different land use watersheds.

Based on the results of the Pearson correlation analysis (Table 5), there is no correlation between the *FF*_*40*_ and rainfall runoff and watershed characteristics, except *I*_*max5*,_ *ADD* and *R*_*D*_. The *FF*_*40*_ of the pollutant concentrations (*COD r = 0*.*462*, *TSS r = 0*.*486*, *TN r = 0*.*416*, *TP r = 0*.*394) (P<0*.*05*) are positively and strongly correlated to the maximum rainfall intensity. These results are same as those of previous studies, which were also carried out in urban catchments [26, 27]. The magnitude of the first flushes of *COD* and *TN* were correlated to the *ADD*. The rain events with longer antecedent dry weather conditions were more likely to result in a higher first flush. Where anthropogenic factors (such as motor vehicle traffic volume, overall “wear and tear” of the drainage area between storm events and poor litter management) are available, they contribute to first flush pollutant loading (e.g., *COD* and *TN*). Non first flush pollutants (*TSS* and *TP*) do not have excess buildup of pollutants due to external factors, such as solids from bare land and tire interaction, and are driven largely by storm characteristics [28].

Previous studies concluded that *EMCs* and first flush are complex and site specific [9, 11, 27]. In comparison with the results for other urban catchments reported by the above studies, the magnitude of the *EMCs* found in the current work is bigger, but the first flush is not stronger in the Tongsha reservoir watershed. Further, the *FF*_*40*_ in the current study does not spread over a wide range around the mean values, as seen in the standard deviation coefficients and variation coefficients. These results can be explained by the fact that Tongsha reservoir catchment is located in a wet region characterized by high intensity rain events. Bare land ready for construction and poor litter management might both have significant effects on *EPLs* and *FF*_*40*_. This means that other catchment characteristics, such as urban form and environmental management, have important roles in influencing pollutant wash-off, rather than this being dominated by the impervious area fraction only. All of these factors resulted in the discharge patterns of stormwater runoff being more complex in the catchments in the rapidly industrialized city examined in this work than those from the other areas.

**Fig 5:**
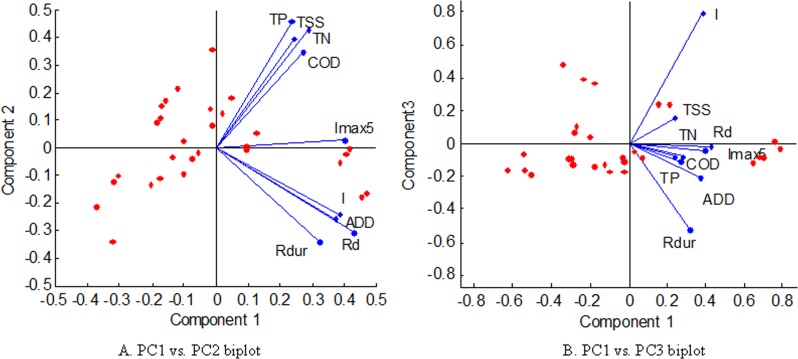
*PCA* biplots for the *FF*_*40*_ dataset.

Fig. 5 shows the results of *PCA* biplots, which consist of Fig. 5-A and Fig. 5-B. Fig. 5-A displays the *PC1* vs. *PC2* biplot and Fig. 5-B shows the *PC1* vs. *PC3* biplot. Based on Fig. 5, the *I*_*max5*_ vector indicates a relatively close correlation with all pollutant *FF*_*40*_ in Fig. 5-A and Fig. 5-B, while the *ADD* and *R*_*D*_ vector only indicates a close correlation with *COD* and *TN*. These observations confirm that *I*_*max5*_ can play a more influential role in the first flush than the *ADD* and *R*_*D*_.

Table 6 presents the multiple linear regression results to estimate the pollution loads of *FF*_*40*_. *ADD* shows significant relationships with the pollution loads of *FF*_*40*_ in all the regression models. Variables *I* are also important for *TSS* and *TP* models in all three different land use watersheds. The model of TP is the most special parameter, which is affected by *R*_*D*_, *I* and *ADD*.

**Table 6:** Multiple linear regression results for the storm pollution loads of *FF*_*40*_ (*|FF*_*40*_ *|*) of Tongsha reservoir watershed.

		*R*_*D*_	*R*_*Dur*_	*I*	*I*_*max5*_	*ADD*		
	*a*	*b*	*c*	*d*	*e*	*f*	*R* ^*2*^	*p*
*COD*	37.430	1.032	0.254	2.292	13.081	0.359*	0.593	0.051
*TSS*	45.463	1.253	0.308	2.784*	15.888	4.360**	0.633	0.023
*TN*	2.043	0.056	0.014	0.125	0.714	0.020*	0.605	0.041
*TP*	0.513	0.014*	0.003	0.031*	0.179	0.005*	0.623	0.029

*I*_*max5*_: Max 5-min intensity,

*I*: Mean rainfall intensity,

*R*_*Dur*_: Rainfall duration,

*R*_*D*_: Rainfall depth,

*ADD*: Antecedent dry days.

**: *P* values<0.01,

*: *P* values <0.05.

Based on the above observations, *I*_*max5*_, *I* and *IMR* are the critical parameters affecting the urban rainfall runoff pollutant event mean concentrations (*EMCs*). The critical parameters for *FF*_*40*_ are *I*_*max5*_, *ADD* and *R*_*D*_. For the stormwater |*FF*_*40*_ *|*, the results of multiple linear regression analysis with regard to rainfall characteristics show that *I*_,_ *ADD* and *R*_*D*_ are the critical parameters.

## Conclusions

The study of non-point source pollution is essential for rapidly industrialized urban areas and the successful operation of National Water Pollution Control and Management Major Projects in China. The characteristics of rainfall pollutant migration were analyzed in Tongsha reservoir catchment in the current study. Mass first flush ratio *(MFFn)* was used to identify the first flush effect. This study obtained new insights about the effects of rainfall characteristics on runoff pollutant wash-off, event mean concentrations (*EMCs*), event pollution load per unit area (*EPLs*) and first flush of 40% (*FF*_*40*_), suggesting both the principal influential factors and the appropriate treatment criteria. The following conclusions are made based on the results of this work:

The pollutant wash-off process of runoff during rainfall events has significant temporal and spatial variations. The pollutant concentrations peaked about 10 to 40 min later than the rainfall intensity peak, although the exact time depended on the catchment characteristics. The variability of the pollutant wash-off characteristics within the same storm event is significantly influenced by both pollutant species and watershed land-use.

According to the results of the first flush effect dimensionless cumulative and *MFFn* analysis, the first flush effect appears in the order of Niushan (*NS*) industrial zone> Dalingshan (*DLS*) mixed commercial and residential area> Tongsha (*TS*) parking lot. The first flush effect will be more distinct when the event has greater max 5-min intensity (*I*_*max5*_)_,_ rainfall depth (*R*_*D*_) and antecedent dry days (*ADD*) values.

Event mean concentrations (*EMCs*) and event pollution load per unit area (*EPLs*) were widely distributed because of the various characteristics of the sites examined in this work. The Dalingsha residential and commercial catchment had the highest median *EMCs* and *EPLs* for *BOD*, *COD*, *TSS*, *NH*_*4*_^*+*^ *-N*, and *TP*, followed by the Niushan industrial and parking catchments. In contrast, the Niushan industrial catchment had the highest median *EMCs* and *EPLs* for *Fe*, *Zn* and *Cu*. The results of this work confirm that in the Tongsha catchment mean rainfall intensity (*I*), Impervious rate (*IMR*) and Max 5-min intensity (*I*_*max5*_) are the critical parameters that influence *EMCs*, while *I*_*max5*_, *ADD* and *R*_*D*_ are the critical parameters of *FF*_*40*_, and *I*, *ADD* and *R*_*D*_ are the critical parameters influencing |*FF*_*40*_ *|*. It should be noted that the paper is limited to the analysis of temporal variations in pollutant wash-off during rain events and the spatial variations with regard to different land-use catchments, quantitative discussions in the case study are site-specific. The temporal and spatial characteristics of runoff pollution in a rapidly industrialized urban, particularly for heavy storm, require further study.

## Supporting Information

S1 File Contains the following files. **Table A.** Stormwater event mean concentrations (*EMCs*) data for Dalingshan catchment. **Table B.** Stormwater event mean concentrations (*EMCs*) data for Niushan catchment. **Table C.** Stormwater event mean concentrations (*EMCs*) data for Tongsha catchment. **Table D.** Stormwater event pollution loads per unit area (*EPLs*) data for Dalingshan catchment. **Table E.** Stormwater event pollution loads per unit area (*EPLs*) data for Niushan catchment. **Table F.** Stormwater event pollution loads per unit area (*EPLs*) data for Tongsha catchment. **Table G.** Stormwater pollutant loads rate transported by the first 40% of runoff volume (*FF40*) data for DalingshanTongsha catchment. **Table H.** Stormwater pollutant loads rate transported by the first 40% of runoff volume (*FF40*) data for Niushan catchment. **Table I.** Stormwater pollutant loads rate transported by the first 40% of runoff volume (*FF40*) data for Tongsha catchment.
(PDF)
