# Supplementary material for: Stormwater Runoff Pollutant Loading Distributions and Their Correlation with Rainfall and Catchment Characteristics in a Rapidly Industrialized City
Source: PLoS One. 2015 Mar 16;10(3):e0118776. doi: 10.1371/journal.pone.0118776 (PMC4361324; doi:10.1371/journal.pone.0118776)
Supplement: S1 File — Table A. Stormwater event mean concentrations (EMCs) data for Dalingshan catchment. Table B. Stormwater event mean concentrations (EMCs) data for Niushan catchment. Table C. Stormwater event mean concentrations (EMCs) data for Tongsha catchment. Table D. Stormwater event pollution loads per unit area (EPLs) data for Dalingshan catchment. Table E. Stormwater event pollution loads per unit area (EPLs) data for Niushan catchment. Table F. Stormwater event pollution loads per unit area (EPLs) data for Tongsha catchment. Table G. Stormwater pollutant loads rate transported by the first 40% of runoff volume (FF40) data for DalingshanTongsha catchment. Table H. Stormwater pollutant loads rate transported by the first 40% of runoff volume (FF40) data for Niushan catchment. Table I. Stormwater pollutant loads rate transported by the first 40% of runoff volume (FF40) data for Tongsha catchment. (PDF) [file pone.0118776.s001.pdf]

SUPPORTING INFORMATION

Stormwater Runoff Pollutant Loading Distributions and Their Correlation with Rainfall and Catchment Characteristics in a Rapidly Industrialized City

Dongya Li [1], Jinquan Wan [1,2,*], Yongwen Ma [1,3], Yan Wang [1], Mingzhi Huang [1] and Yangmei Chen [1]

[1] College of Environment and Energy, South China University of Technology, Guangzhou 510006, China;

[2] The Key Lab of Pollution Control and Ecosystem Restoration in Industry Clusters, Ministry of Education, Guangzhou 510006, China

[3] State Key Laboratory of Pulp and Paper Engineering, South China University of Technology, Guangzhou 510640, China

***** Author to whom correspondence should be addressed; E-Mail: ppjqwan@scut.edu.cn; Tel./Fax: +86-020-87114970.

Tables

Table A. Stormwater event mean concentrations (*EMCs*) data for Dalingshan catchment.

Table B. Stormwater event mean concentrations (*EMCs*) data for Niushan catchment.

Table C. Stormwater event mean concentrations (*EMCs*) data for Tongsha catchment.

Table D. Stormwater event pollution loads per unit area (*EPLs*) data for Dalingshan catchment.

Table E. Stormwater event pollution loads per unit area (*EPLs*) data for Niushan catchment.

Table F. Stormwater event pollution loads per unit area (*EPLs*) data for Tongsha catchment.

Table G. Stormwater pollutant loads rate transported by the first 40% of runoff volume (*FF40*) data for DalingshanTongsha catchment.

Table H. Stormwater pollutant loads rate transported by the first 40% of runoff volume (*FF40*) data for Niushan catchment.

Table I. Stormwater pollutant loads rate transported by the first 40% of runoff volume (*FF40*) data for Tongsha catchment.

Table A. Stormwater event mean concentrations (*EMCs*) data for Dalingshan catchment

Date	*COD*	*TSS*	*TN*	*TP*	NH_4^+*-N*	*Fe*	*Zn*
15/4/2009	277.44	416.01	15.23	3.87	7.75	2.98	0.57
15/9/2009	287.75	352.36	3.67	1.09	1.79	0.33	0.22
20/10/2009	103.25	194.15	6.29	0.42	5.10	1.8	0.41
2/6/2010	121.21	141.03	3.49	1.72	1.35	0.12	0.12
28/6/2010	467.63	516.49	20.42	4.74	5.71	2.34	0.43
20/10/2010	395.83	297.34	13.53	2.69	8.60	2.61	0.3
16/4/2011	334.97	395.63	39.05	3.81	9.13	1.54	0.11
3/5/2011	144.32	187.62	5.03	0.75	3.51	0.78	0.35
9/8/2011	328.55	462.83	27.02	7.09	6.04	1.32	0.44
21/9/2011	567.11	708.40	33.22	5.55	6.25	1.77	0.38

Table B. Stormwater event mean concentrations (*EMCs*) data for Niushan catchment

Date	*COD*	*TSS*	*TN*	*TP*	NH_4^+-*N*	*Fe*	*Zn*	*Cu*
15/4/2009	291.33	337.89	13.18	3.65	7.49	6.78	4.78	0.47
15/9/2009	164.48	216.10	2.46	0.86	1.71	1.3	2.10	0.13
20/10/2009	92.04	134.07	4.86	0.67	3.72	4.1	3.60	0.37
2/6/2010	270.68	184.10	6.45	1.39	0.99	1.23	1.80	0.39
28/6/2010	331.28	427.55	13.97	4.06	4.56	5.4	4.80	0.21
20/10/2010	106.09	435.83	6.01	1.42	5.08	4.54	1.57	0.33
16/4/2011	187.11	239.06	9.05	2.26	7.09	6	5.07	0.11
3/5/2011	203.36	258.25	5.30	2.15	4.77	3.7	3.43	0.41
9/8/2011	81.93	96.93	16.65	1.48	2.62	5.89	2.70	0.38
21/9/2011	486.22	651.30	11.88	3.24	4.72	3.76	5.12	0.29

Table C. Stormwater event mean concentrations (*EMCs*) data for Tongsha catchment

Date	*COD*	*TSS*	*TN*	*TP*	NH_4^+-*N*	*Fe*
15/4/2009	67.97	107.68	2.78	0.98	1.00	0.85
15/9/2009	56.90	58.20	1.51	0.43	0.45	0.22
20/10/2009	71.36	92.59	1.88	0.32	0.93	0.51
2/6/2010	24.41	25.80	3.32	0.43	0.12	0.13
28/6/2010	81.17	96.76	1.45	1.79	1.19	0.46
20/10/2010	122.21	133.09	1.22	0.28	1.36	0.58
16/4/2011	106.02	117.83	3.32	0.09	1.08	0.33
3/5/2011	71.31	84.26	0.91	1.52	0.98	0.15
9/8/2011	17.18	78.11	4.34	0.28	0.37	0.11
21/9/2011	60.86	72.87	2.60	4.05	1.05	0.31

Table D. Stormwater event pollution loads per unit area (*EPLs*) data for Dalingshan catchment

Date	*COD*	*TSS*	*TN*	*TP*	NH_4^+*-N*	*Fe*	*Zn*
15/4/2009	130.92	196.30	7.19	1.83	3.66	1.41	0.27
15/9/2009	25.29	30.97	0.32	0.10	0.16	0.03	0.02
20/10/2009	8.08	15.19	0.49	0.03	0.40	0.14	0.03
2/6/2010	9.10	10.59	0.26	0.13	0.10	0.01	0.01
28/6/2010	126.09	139.27	5.50	1.28	1.54	0.63	0.12
20/10/2010	132.15	99.26	4.52	0.90	2.87	0.87	0.10
16/4/2011	33.87	40.00	3.95	0.39	0.92	0.16	0.01
3/5/2011	11.63	15.12	0.41	0.06	0.28	0.06	0.03
9/8/2011	55.99	78.87	4.60	1.21	1.03	0.22	0.07
21/9/2011	38.23	47.75	2.24	0.37	0.42	0.12	0.03

Table E. Stormwater event pollution loads per unit area (*EPLs*) data for Niushan catchment

Date	*COD*	*TSS*	*TN*	*TP*	NH_4^+*-N*	*Fe*	*Zn*	*Cu*
15/4/2009	138.30	160.40	6.26	1.73	3.55	3.22	2.27	0.22
15/9/2009	16.22	21.31	0.24	0.08	0.17	0.13	0.21	0.01
20/10/2009	7.24	10.55	0.38	0.05	0.29	0.32	0.28	0.03
2/6/2010	20.45	13.91	0.49	0.11	0.07	0.09	0.14	0.03
28/6/2010	89.86	115.98	3.79	1.10	1.24	1.46	1.30	0.06
20/10/2010	35.63	146.37	2.02	0.48	1.71	1.52	0.53	0.11
16/4/2011	19.03	24.32	0.92	0.23	0.72	0.61	0.52	0.01
3/5/2011	16.60	21.09	0.43	0.18	0.39	0.30	0.28	0.03
9/8/2011	14.04	16.61	2.85	0.25	0.45	1.01	0.46	0.07
21/9/2011	32.97	44.17	0.81	0.22	0.32	0.25	0.35	0.02

Table F. Stormwater event pollution loads per unit area (*EPLs*) data for Tongsha catchment

Date	*COD*	*TSS*	*TN*	*TP*	NH_4^+-*N*	*Fe*
15/4/2009	19.14	30.33	0.78	0.27	0.28	0.24
15/9/2009	4.00	4.10	0.11	0.03	0.03	0.02
20/10/2009	3.33	4.32	0.09	0.02	0.04	0.02
2/6/2010	1.09	1.16	0.15	0.02	0.01	0.01
28/6/2010	13.06	15.57	0.23	0.29	0.19	0.07
20/10/2010	24.35	26.52	0.24	0.06	0.27	0.12
16/4/2011	6.40	7.11	0.20	0.01	0.07	0.02
3/5/2011	5.55	6.55	0.07	0.12	0.08	0.01
9/8/2011	2.44	11.12	0.62	0.04	0.05	0.02
21/9/2011	2.45	2.93	0.10	0.16	0.04	0.01

Table G. Stormwater pollutant loads rate transported by the first 40% of runoff volume (*FF_{40}*) data for DalingshanTongsha catchment

Date	*COD*	*TSS*	*TN*	*TP*	*NH_4^+-N*
15/4/2009	0.62	0.66	0.73	0.66	0.87
15/9/2009	0.50	0.55	0.58	0.72	0.58
20/10/2009	0.49	0.54	0.64	0.63	0.57
2/6/2010	0.48	0.48	0.53	0.57	0.51
28/6/2010	0.56	0.58	0.70	0.70	0.61
20/10/2010	0.58	0.65	0.71	0.63	0.79
16/4/2011	0.57	0.64	0.80	0.75	0.67
3/5/2011	0.47	0.41	0.38	0.50	0.43
9/8/2011	0.60	0.63	0.52	0.76	0.66
21/9/2011	0.51	0.50	0.58	0.58	0.53

Table H. Stormwater pollutant loads rate transported by the first 40% of runoff volume (*FF_{40}*) data for Niushan catchment

Date	*COD*	*TSS*	*TN*	*TP*	*NH_4^+-N*
15/4/2009	0.66	0.65	0.65	0.75	0.69
15/9/2009	0.49	0.64	0.49	0.77	0.67
20/10/2009	0.47	0.60	0.63	0.54	0.63
2/6/2010	0.54	0.47	0.55	0.70	0.50
28/6/2010	0.57	0.56	0.55	0.72	0.59
20/10/2010	0.57	0.46	0.37	0.53	0.48
16/4/2011	0.54	0.64	0.55	0.70	0.68
3/5/2011	0.40	0.39	0.32	0.37	0.41
9/8/2011	0.55	0.66	0.62	0.53	0.70
21/9/2011	0.40	0.64	0.32	0.37	0.67

Table I. Stormwater pollutant loads rate transported by the first 40% of runoff volume (*FF_{40}*) data for Tongsha catchment

Date	*COD*	*TSS*	*TN*	*TP*	*NH_4^+-N*
15/4/2009	0.61	0.54	0.76	0.70	0.57
15/9/2009	0.58	0.55	0.70	0.70	0.58
20/10/2009	0.69	0.65	0.79	0.79	0.52
2/6/2010	0.48	0.56	0.56	0.58	0.56
28/6/2010	0.58	0.53	0.71	0.54	0.59
20/10/2010	0.37	0.32	0.36	0.31	0.34
16/4/2011	0.61	0.58	0.66	0.63	0.61
3/5/2011	0.38	0.46	0.43	0.44	0.48
9/8/2011	0.52	0.49	0.66	0.61	0.69
21/9/2011	0.47	0.50	0.53	0.52	0.53
